# The Participation and Environment Measure—Children and Youth (PEM‐CY): Cultural Adaptation, Validity, and Reliability of the Persian Version for 5‐ to 17‐Year‐Old Children With Cerebral Palsy

**DOI:** 10.1155/oti/3590445

**Published:** 2026-05-04

**Authors:** Marzieh Pashmdarfard, Sara Zamiran, Navid Mirzakhani, Ebrahim Mahmoudi

**Affiliations:** ^1^ Department of Occupational Therapy, School of Rehabilitation Sciences, Imam Hossein Hospital, Shahid Beheshti University of Medical Sciences, Tehran, Iran, sbmu.ac.ir; ^2^ Department of Occupational Therapy, School of Rehabilitation, Shahid Beheshti University of Medical Sciences, Tehran, Iran, sbmu.ac.ir; ^3^ Department of Occupational Therapy, School of Rehabilitation Sciences, Shahid Beheshti University of Medical Sciences, Tehran, Iran, sbmu.ac.ir; ^4^ School of Physical and Occupational Therapy, McGill University, Montreal, Quebec, Canada, mcgill.ca

**Keywords:** cerebral palsy, children, environment, participation, reliability, validation

## Abstract

**Background:**

Participation in daily activities is essential for health and well‐being. It helps in physical, mental, and emotional health and plays a significant role in the positive development of children and young people. As it is a crucial goal for health and rehabilitation services, the purpose of this study was to translate and assess the psychometric properties of the Persian version of the Participation and Environment Measure—Children and Youth (PEM‐CY) in 5‐ to 17‐year‐old children with cerebral palsy.

**Method:**

The study involved 150 parents of children with CP (n = 100) and typically developing peers (n = 50), aged 5–17 years. To evaluate face and content validity, we consulted occupational therapists and parents of children with CP. Moreover, the Mann–Whitney test used to assess divergent validity. Internal consistency was assessed using Cronbach′s alpha (*α*), whereas test‐retest reliability was evaluated using intraclass correlation coefficients (ICCs).

**Results:**

The internal consistency (0.77–0.95) and test‐retest reliability (0.85–0.95) of various summary scores ranged from moderate to very strong. The divergent validity of the Persian version of PEM‐CY was supported by significant differences between children with and without disabilities on the participation and environment scales (p < 0.05).

**Conclusions:**

The Persian version of the PEM‐CY is a valid and reliable tool to determine the participation and environmental factors in the home, at school, and in community settings in Persian children and youth aged 5–17 years, with CP.

**Summary**

• This study evaluated the Persian version of the Participation and Environment Measure for Children and Youth (PEM‐CY), highlighting its significance for health and well‐being, especially for children with cerebral palsy (CP).
• Involving 150 parents of children aged 5 to 17—100 with CP and 50 typically developing—findings revealed strong internal consistency and test‐retest reliability for the tool.
• Significant differences in participation and environment scores were observed between children with and without disabilities, confirming its discriminant validity.
• Although some age‐related differences were noted, they were inconsistent.
• Overall, the Persian PEM‐CY is a reliable measure for assessing participation and environmental factors in this population.

## 1. Introduction

Participation in daily activities plays a crucial role in ensuring health and well‐being [1, 2]. Furthermore, it is a key process contributing to the positive developmental outcomes among children and young people and serves as a major goal for health and rehabilitation services [3]. Participation is a complex multidirectional construct influenced by social, attitudinal, and physical aspects of the environment [4]. A growing body of literature reveals that children with CP, the most prevalent cause of physical disability in childhood, and other physical disabilities exhibit significantly lower participation rates in activities across various environments, including home, school, and community settings [5–7]. These participation restrictions often extend into adulthood, highlighting the need for early and sustained intervention. Adequate support for families during childhood, the provision of timely and targeted interventions, and efforts to enhance these children′s autonomy are crucial measures to mitigate participation restrictions [4, 8].

The International Classification of Functioning, Disability and Health: Children and Youth Version (ICF‐CY) defines participation as “involvement in a life situation,” which, for children, means participating in activities at home, in school, and in the community [1]. Studies consistently indicate that children with physical disabilities face significantly greater limitations in their participation compared with their typically developing peers [9–12]. They often engage in a narrower range of activities, which tends to skew toward those that are less physically and socially demanding, such as household chores [13–15]. Participation in children with physical disabilities is more restricted than that of their peers with other disabilities, and they are at risk of occupational deprivation [16, 17].

Contemporary theory highlights that participation is the result of dynamic interactions between individuals and their environments. Therefore, sound measures of each of these key constructs will be needed to improve our understanding of their complex interrelationships [3, 18]. Research indicates that personal factors, including age, gender, and executive functions, along with environmental factors such as accessibility, socioeconomic status, cultural background, and supportive environments, significantly influence children′s participation patterns in various activities [19, 20]. These factors collectively shape how children engage in their surroundings and contribute to differences in participation patterns across different countries [11].

In recent years, innovative measurement tools have been developed to facilitate the evaluation of participation in specific environments. These instruments have been designed for and applied to various purposes, including clinical assessment, outcome tracking, and research endeavors. Despite these advancements, none of the current measures fully meet the criteria for broad ICF‐CY coverage, age inclusivity, and feasibility for large‐scale population research. The limitations of various assessments for children′s participation and enjoyment are significant. Tools such as the Children′s Assessment of Participation and Enjoyment (CAPE), School Function Assessment (SFA), and Assessment of Life Habits for Children (LIFE‐H) are hindered by their length, affecting their practical use. Additionally, instruments like the Child and Adolescent Scale of Participation and SFA focus on specific populations, limiting their generalizability. Lastly, the child participation questionnaire has a narrow age range, further constraining its applicability. These factors are essential to consider when selecting assessment tools [3, 21].

The PEM‐CY was developed by Coster using the theoretical framework of the ICF‐CY and through extensive conceptual collaboration with parents of children both with and without disabilities. The PEM‐CY provides comprehensive data on the participation patterns of children and young people, as well as the environmental factors affecting their participation in daily activities. Unlike other tools, it evaluates both participation and environmental factors across home, school, and community settings. The PEM‐CY also captures attendance and involvement, and differentiates participation and environment among various groups, such as children with and without disabilities, and families with different income levels [3]. According to the definition of participation within the ICF‐CY, it is essential to employ thorough assessment tools that can be tailored to the local culture and evaluate children′s participation across various life contexts [22].

Recognizing the critical significance of this tool for evaluating cultural and environmental factors and noting the lack of a Persian version, this study intends to translate, culturally adapt, and rigorously assess the reliability and validity of the Persian version of the PEM‐CY for children aged 5–17 with CP in Iran.

## 2. Materials and Methods

### 2.1. Study Design

The development of the Persian language version of the PEM‐CY was completed in two phases that at the first phase, the English version was translated and cross‐cultural adaptation was performed. The second phase assessed face, content, and construct validity for validity and internal consistency and test‐retest reliability for the Persian version of PEM‐CY. This study was done between September 2024 and March 2025 in Tehran, Iran.

### 2.2. Translation and Cross‐Cultural Adaptation

In the present study, the original version of PEM‐CY was purchased from Shop CanChild by the corresponding author, and permission for the translation and validation of PEM‐CY was obtained from the CanChild group. The translation process was conducted using the International Quality of Life Assessment (IQOLA) method, including forward and backward translation [23]. First, two independent translators with expertise in rehabilitation translated the original questionnaire from English into Persian (forward translation). These two versions were then synthesized into a single version by the research team. At this stage, some activities were culturally adapted to better suit the Iranian context. Subsequently, a third bilingual translator performed a back‐translation into English. The back‐translated version was compared with the original questionnaire by the authors and clinical experts to ensure conceptual equivalence.

The cultural adaptation of the translated version was verified during translation and assessed by an expert panel regarding the examples mentioned for activities, with no inconsistencies found. The final Persian version was confirmed by the PEM‐CY team, by Rachel Teplicky, CanChild′s Business and Engagement Officer.

### 2.3. Validity

#### 2.3.1. Content Validity

The content validity was calculated based on the content validity index (CVI) and the content validity ratio (CVR) method. CVR or Lawshe′s method is a quantitative method for determining the content validity and is widely accepted. In this method, 15 occupational therapists with at least 5 years of experience in the field of pediatric occupational therapy assessed the content validity of the Persian version of PEM‐CY. CVI or Waltz and Bausell method is an approach to assess validity. In this method, each item needs a score of more than 0.79 to be accepted [24].

From these 15 experts, eight were the PhD faculty members, four were PhD students, and three had a master′s degree.

#### 2.3.2. Face Validity

The face validity was tested by asking 20 parents of children with CP. A 5‐point Likert scale was used to assess relevance, understanding of concepts, proportion, and clarity of the questionnaire.

#### 2.3.3. Construct Validity

Construct validity is a criterion of a significant amount of a scale in practical application or determines whether the scale measures what it is considering [25]. In the present study, divergent validity was used to evaluate the construct validity of the Persian version of PEM‐CY. The COSMIN checklist suggests a minimum sample size of over 50 participants for internal consistency and more than 50 per group for known‐groups validity [26].

#### 2.3.4. Divergent Validity

Fifty parents of children with CP and 50 parents of typically developing children were asked to complete the Persian version of PEM‐CY to compare the participation of children with CP and their typically developing peers. If the correlation coefficient is between 0.00 and 0.19, the divergent validity is very weak; if it is between 0.20 and 0.39, the divergent validity is weak; if it is between 0.40 and 0.59, the divergent validity is moderate; if it is between 0.60 and 0.79, the divergent validity is strong; and if it is above 0.80, the divergent validity is very strong [27].

### 2.4. Reliability

#### 2.4.1. Internal Consistency

In order to investigate the internal consistency of the Persian version of PEM‐CY in children with CP, 100 parents of children with CP completed the questionnaire, and the internal consistency of the items was examined through the Cronbach′s alpha coefficient. If the coefficient is greater than 0.8, the internal consistency in that tool or subtest is at a high level. The values of 0.7–79 are moderate level and values lower than 0.7 indicate a low level of internal consistency [27].

#### 2.4.2. Test‐Retest Reliability

We used the intraclass correlation coefficient (ICC) to measure test‐retest reliability. The questionnaires were given to 40 parents who agreed to cooperate with the researcher again after a 2‐week interval. If the coefficient is less than 0.4 (ICC < 0.4), reliability is poor; between 0.4 and 0.7 (0.4 < ICC < 0.7), reliability is fair to good; and when it is above 0.7 (ICC > 0.7), it indicates excellent reliability [27].

#### 2.4.3. Statistical Analysis

The results of the present study were analyzed using SPSS Version 26. Before performing the main analyses, the normality of the research variables was tested using the Kolmogorov–Smirnov test, which did not confirm the assumption of normality of the research variables (p ≤ 0.05). Descriptive analyses were used to explain the characteristics of children and their parents. Therefore, to examine the divergent validity, nonparametric Mann–Whitney U tests were used to compare PEM‐CY scores between groups. Test‐retest reliability and internal consistency were assessed using the ICC and Cronbach′s alpha.

### 2.5. Participants

Given that the PEM‐CY questionnaire comprises 60 items, adherence to psychometric research principles dictates that the sample size should be at least equal to or double the number of test items. Consequently, a total of 100 participants were employed in this assessment to ensure robust analysis and statistical validity [28, 29].

Children were included in the study if (a) they and their mothers were fluent in the Persian language, (b) they were between 5 and 17 years old, (c) they did not have cognitive problems or a SPARKLE test score ≤ 70. We exclude participants who were not eager to cooperate in the study.

### 2.6. Measurements

#### 2.6.1. Demographic Information Questionnaire

The demographic information questionnaire was a researcher‐developed questionnaire designed to gather crucial data on participants′ age, sex, response to the questionnaire, marital status, educational attainment, and type of CP.

#### 2.6.2. PEM‐CY

The PEM‐CY is a parent‐report questionnaire to assess participation and environment factors in the home, at school, and within community settings [3]. The participation sections included 10 activities in the home setting, five activities in the school setting, and 10 in the community setting. For each activity, parents are asked to determine the participation frequency (how frequently has the child participated with eight options: daily to never), participation involvement (how involved the child is while participating the activity rated on a five‐point scale: very involved to minimally involved) and whether change is desired (do the parents want to see change in the child′s participation in this type of activity: no or yes, with five different types of change). After answering the participation section, parents are asked to rate environmental features to identify supports and barriers (e.g., do the features of the environment help or make it harder for the child to participate in activities in home/school/community setting) [18].

##### 2.6.2.1. The SPARKLE Test.

This test assesses the cognitive function of children with CP and movement disorders in 3 levels: below 50, between 50 and 70, and above 70. Based on this test, parents of these children will answer four questions to determine the cognitive function of their children [30, 31].

### 2.7. Procedure

Approval for the present study was obtained by the local ethics committee of the Shahid Beheshti University of Medical Science (IR.SBMU.RETECH.REC.1401.847). Written informed consent was gathered from all participants in the study. Children with CP and their parents from the different regions of Tehran were included in the study if they met the inclusion criteria.

## 3. Results

### 3.1. Participants

The mean age of children with CP participating in the study was 11.31 ± 5.12 years (minimum age: 5 years, maximum age: 17 years), of which 58 were girls (58%) and 32 were boys (32%). The mean age of parents of children with CP (all mothers) was 36.5 ± 3.11 years. Other demographic information of the participants is shown in Table 1.

**Table 1:** Characteristics of participants.

Variables	With CP *n* = 100	Without CP *n* = 50
Mean age	11.31 (5.12)	9.11 (4.41)
Gender		
Male	42 (42%)	19 (38%)
Female	58 (58%)	31 (62%)
School		
Yes	59 (59%)	42 (84%)
No	41 (41%)	8 (16%)
Response to questionnaires		
Mother	100 (100%)	48 (96%)
Father	0	2 (4%)
Marital status of responders		
Married	87 (87%)	41 (82%)
Divorced	12 (12%)	7 (14%)
Single	1 (1%)	2 (4%)
Type of CP		
Hemiplegia	35 (35%)	—
Diplepia	45 (45%)	—
Quadriplegia	13 (13%)	—
Ataxia	2 (2%)	—
Athetoid	5 (5%)	—

### 3.2. Translation

The back translation was sent via email to the CanChild review team, and the translated version was approved. During the cultural adaptation process, there was no need for further changes due to cultural differences, and all items were culturally relevant. Only examples mentioned for activities were translated to the culture. After approval, the final Persian version was reviewed for face and content validity.

#### 3.2.1. Face Validity

In the face validity assessment process, the results showed that all items of the PEM‐CY questionnaire were acceptable to parents and occupational therapists and did not cause any particular problems for the respondents to understand, and all parents of children with CP (20/20) and all occupational therapists (15/15) found the Persian version of the PEM‐CY to be appropriate in terms of translation quality, comprehensibility, and adequacy for the Iranian society.

#### 3.2.2. Content Validity

Content validity results indicated that all items had high content validity (CVI ≥ 79*%*), and there was no item that did not obtain the required score.

#### 3.2.3. Divergent Validity

Table 2 shows the PEM‐CY participation and environment scores for the groups of children with CP and their TD peers. The results show that in all PEM‐CY domains, children with CP showed poorer scores than their TD peers. Regarding the participation domain, we found significant differences (p < 0.01) between groups for participation and number of activities performed in all environments, frequency of participation in the community, and adequate change in school. Regarding the environment domain, we found significant differences (p < 0.01) between groups in all domains in the home, school, and community environments.

**Table 2:** Differences between groups in the PEM‐CY domains.

Setting domain	Children with CP	Children without CP	*p* value
**Home**	**Mean (±SD)**	**M (±SD)**	
Number of activities (out of 0)	6.03 (2.30)	9.71 (0.62)	< 0.001 ^∗^
Frequency (out of 7)	6.21 (0.68)	6.46 (0.32)	0.920
Involvement (out of 5)	3.41 (1.28)	4.09 (0.66)	0.007 ^∗^
Desire for change (%)	58.08 (31.21)	53.01 (28.13)	0.304
Supports (%)	69.01 (20.49)	81.77 (17.14)	0.001 ^∗^
Barriers (%)	31.59 (20.72)	19.23 (16.24	0.002 ^∗^
Environmental helpfulness (%)	88.86 (11.63)	96.38 (7.79)	0.002 ^∗^
Environmental resources (%)	81.40 (13.67)	89.57 (11.53)	0.002 ^∗^
Overall environmental support (%)	84.71 (10.85)	92.38 (8.11)	< 0.001 ^∗^
**School**			
Number of activities (out of 5)	2.79 (1.72)	4.13 (1.15)	< 0.001 ^∗^
Frequency (out of 7)	4.03 (2.26)	4.77 (2.26)	0.167
Involvement (out of 5)	2.96 (1.78)	4.12 (1.13)	< 0.001 ^∗^
Desire for change (%)	56.38 (37.71)	33.26 (31.07)	0.004 ^∗^
Supports (%)	55.50 (31.36)	84.62 (18.09)	< 0.001 ^∗^
Barriers (%)	29.89 (24.64)	12.82 (11.75)	< 0.001 ^∗^
Environmental helpfulness (%)	72.22 (32.42)	92.78 (16.37)	< 0.001 ^∗^
Environmental resources (%)	70.01 (32.23)	90.81 (16.56)	< 0.001 ^∗^
Overall environmental support (%)	72.34 (30.71)	91.86 (16.00)	< 0.001 ^∗^
**Community**			
Number of activities (out of 10)	4.76 (2.09)	6.76 (2.01)	< 0.001 ^∗^
Frequency (out of 7)	2.02 (1.10)	2.80 (0.96)	< 0.001 ^∗^
Involvement (out of 5)	1.86 (1.16)	2.75 (1.02)	< 0.001 ^∗^
Desire for change (%)	58.23 (32.21)	38.97 (27.80)	< 0.001 ^∗^
Supports (%)	51.21 (23.50)	72.24 (16.41)	< 0.001 ^∗^
Barriers (%)	47.59 (23.51)	25.60 (16.33)	< 0.001 ^∗^
Environmental helpfulness (%)	74.94 (16.13)	91.28 (7.82)	< 0.001 ^∗^
Environmental resources (%)	73.66 (14.76)	81.03 (12.19)	0.010 ^∗^
Environmental helpfulness (%)	74.13 (13.60)	81.33 (8.57)	< 0.001 ^∗^

^∗^Statistically significant difference according to the Mann–Whitney U test.

### 3.3. Internal Consistency

Cronbach′s alpha for home indicates a high level of internal consistency in both participation and environment domains, which was between 0.8 and 0.89 for participation and 0.86–0.9 for environment. In the school setting Cronbach′s alpha indicated a high level of internal consistency for the participation domain (0.82–0.83) and moderate to high for environment (0.77–0.9). In the community setting Cronbach′s alpha indicated a moderate to high level of internal consistency for the participation domain (0.78–0.85) and a high level for environment (0.88–0.95). (Table 3).

**Table 3:** Internal consistency and test‐retest reliability values of the PEM‐CY domains.

Setting		Domain	Items	Cronbach′s alpha coefficient (*α*) (*n* = 100)	Intraclass correlation coefficient (ICC) (*n* = 40)
**Home**	**Participation**	Frequency	10	0.80	0.90
		Involvement	10	0.87	0.91
		Desire for change	10	0.89	0.90
	**Environment**	Supports	12	0.86	0.88
		Barriers	12	0.80	0.88
		Environmental helpfulness	7	0.90	0.90
		Environmental resources	5	0.91	0.91
		Overall environmental support	12	0.90	0.88
**School**	**Participation**	Frequency	5	0.82	0.85
		Involvement	5	0.83	0.88
		Desire for change	5	0.82	0.89
	**Environment**	Supports	17	0.77	0.80
		Barriers	17	0.88	0.89
		Environmental helpfulness	9	0.88	0.90
		Environmental resources	8	0.91	0.91
		Overall environmental support	17	0.90	0.90
**Community**	**Participation**	Frequency	10	0.78	0.90
		Involvement	10	0.80	0.88
		Desire for change	10	0.85	0.89
	**Environment**	Supports	16	0.88	0.87
		Barriers	16	0.90	0.85
		Environmental helpfulness	9	0.95	0.95
		Environmental resources	7	0.95	0.91
		Overall environmental support	16	0.95	0.92

### 3.4. Test‐Retest Reliability

The results in Table 3 indicated excellent reliability in all settings and all domains of PEM‐CY. The reliability for the participation domain in settings was between 0.85 and 0.91, and for the environment domain was between 0.87 and 0.92.

## 4. Discussion

The present study is aimed at translating the PEM‐CY into Persian and to evaluate the psychometric properties of its Persian version among children aged 5–17 years with CP. The findings indicate that the Persian version of the PEM‐CY is a valid and reliable instrument for assessing participation and environmental factors related to engagement in home, school, and community activities among Persian‐speaking children, both with and without CP. The findings highlighted significant disparities in both participation and environmental domains, emphasizing the barriers faced by children with CP in various settings.

Validity was established through face, content, and divergent validity assessments, with the results demonstrating high validity across three domains: home, school, and environment. This validates the tool′s effectiveness in discerning differences between children with CP and their typically developing peers.

Analysis of the differences between the two groups reveals that participants with disabilities exhibited lower levels of involvement and engaged in fewer activities across all settings, particularly showing reduced frequency of participation in school and community contexts when compared with their typically developing counterparts. These findings align with measurement properties evaluations of the Chinese, Brazilian, German, Turkish, Korean, Indian, and Spanish versions of the PEM‐CY, all of which similarly documented significant disparities in participation and environmental experiences between children with and without disabilities [4, 18, 32–37]. Interestingly, no significant differences were noted regarding participation frequency at home or the desire for change in that context. This observation is consistent with the original version′s findings, which indicated differences in the perceived desire for change reported by parents of children with and without disabilities, specifically in school and community settings [18]. These findings underscore the challenges that children with CP face in engaging in social, educational, and recreational activities, which are crucial for their overall development and well‐being. This highlighted the need for targeted interventions aimed at enhancing participation.

Our inquiry suggests that parents of children with disabilities tend to maintain an optimistic perspective concerning their children′s potential for increased participation in these contexts. It is broadly acknowledged that opportunities influence participation patterns and the support available in the environment [12, 20]. Consistent with prior research, our findings revealed that caregivers of children with CP perceived a lower level of environmental support, faced more barriers, and reported limited resource availability compared with caregivers of children without disabilities in all settings [4, 18, 32–37]. Differences in supports and barriers also reveal that a substantial portion of children with CP experience fewer available resources and greater hindrances, which can impede their ability to fully participate in activities alongside their peers.

Similar to Turkish, Korean, and Spanish versions, no significant differences were found in environmental supports at home [32, 34, 36]. This result suggests that families provide support at home, regardless of their children′s disabilities. These findings emphasize the necessity of environment‐based interventions to improve the participation of children with disabilities.

This further validates the Persian version of the PEM‐CY as an effective tool for evaluating environmental factors impacting the experiences of Persian children.

The assessment of reliability for the Persian PEM‐CY exhibited strong internal consistency and excellent test‐retest reliability across all domains of the measure. These findings suggest that the items within each domain reflect the same constructs related to participation and environmental factors consistently. The Cronbach′s alpha values indicate that both the participation and environment domains are reliable, affirming the instrument′s usefulness in capturing meaningful data for both clinical and research contexts. High‐internal consistency scores were observed in the domains concerning home and school participation, as well as community environments, corroborating results from the original PEM‐CY and its adaptations in Chinese, Indian, Korean, Turkish, Brazilian, German, and Spanish contexts [37–32, 18, 4].

The significant desire for change reported by children with CP, particularly in the school setting, highlights an important aspect for practitioners in this field. The fact that a majority of participants expressed a desire for increased support in their environments indicates that there is an awareness of how modifications can enhance participation. These insights suggest a critical opportunity for developing individualized strategies that promote inclusion and more accessible and supportive environments for children with disabilities.

The consistency of findings across diverse cultures and locations reinforces the reliability of the PEM‐CY as a measure of participation and environmental factors affecting children and youth.

## 5. Limitations

The present study did not evaluate the differences in children′s participation and the environmental supports or barriers in relation to other significant child or family variables, such as the severity of the disability or family income type. Children with CP who had cognitive impairments and a SPARKLE test score below 70 were excluded from the research due to a focus on environmental barriers. Further research needs to investigate the participation of children with varying cognitive levels. Additionally, a limitation of the study was that all participants were children diagnosed with CP, and children with other movement disorders were not included in the study.

## 6. Conclusion

In conclusion, the present study provides evidence to support the psychometric properties of the Persian version of PEM‐CY for children and youth with/without CP. The Persian PEM‐CY demonstrated good validity and excellent reliability in our study, and it can be used in clinical practice and applied to research to evaluate the participation and environmental factors for children and youth with and without CP.

## Author Contributions

Marzieh Pashmdarfard: formal analysis, methodology. Navid Mirzakhani: supervision. Sara Zamiran: writing – original draft, writing – review & editing. Ebrahim Mahmoudi: data curation.

## Funding

No funding was received for this manuscript.

## Conflicts of Interest

The authors declare no conflicts of interest.

## Data Availability

The data that support the findings of this study are available from the corresponding author upon reasonable request.
